# Dextrose Injections Across Temporomandibular Joint Mobility Disorders: A Scoping Review

**DOI:** 10.3390/jcm15176708

**Published:** 2026-08-29

**Authors:** Julia Kasprzycka, Maciej Chęciński, Justyna Wilk, Wojciech Macek, Maja Kosińska, Amelia Hoppe, Oliwia Jagiełło, Karolina Grzybowska-Kowalczyk, Tomasz Horodniczy, Zuzanna Baniak, Izabella Chyży, Kamila Chęcińska, Maciej Sikora

**Affiliations:** 1 Miodowa Clinic, Głowaccy Medical and Dental Practice Professional Partnership, Kiekrz, Miodowa 2, 62-090 Rokietnica, Poland; lekdentkasprzycka@gmail.com; 2 National Medical Institute of the Ministry of the Interior and Administration, Wołoska 137, 02-507 Warsaw, Poland; maciej.checinski@pimmswia.gov.pl (M.C.); ichyzy@gcm.pl (I.C.); maciej.sikora@pimmswia.gov.pl (M.S.); 3 Department of Maxillofacial Surgery, Hospital of the Ministry of the Interior and Administration, Wojska Polskiego 51, 25-375 Kielce, Poland; 4 Ortosfera Orthodontic Practice, Władysława Reymonta 2, 33-300 Nowy Sącz, Poland; jwilkortosfera@gmail.com; 5 Department of Oral Surgery, Preventive Medicine Center, Komorowskiego 12, 30-106 Cracow, Poland; ld.wojciech.macek@wp.pl (W.M.); majaakosinska@gmail.com (M.K.); amelia.a.hoppe@gmail.com (A.H.); 6 Gabinety Lekarskie Dent-im M.B. Pawelczyk Spółka Jawna, Różana 13/1, 61-577 Poznań, Poland; oliwiajagiellodent@gmail.com; 7 Uśmiech Family Dental Clinic, Nastrojowa 26, 91-496 Łódź, Poland; karolina.orto.gk@gmail.com; 8 Ortho.pl Dental Office, Buforowa 34, 52-131 Wrocław, Poland; tomasz.horodniczy2@gmail.com; 9 Stomatologia Centrum, Moniuszki 3/7, 32-020 Wieliczka, Poland; baniakzuzanna@gmail.com; 10 Department of Biochemistry and Medical Chemistry, Pomeranian Medical University, Powstańców Wielkopolskich 72, 70-111 Szczecin, Poland

**Keywords:** temporomandibular joint, dextrose, prolotherapy, joint hypermobility, recurrent dislocation, disc displacement without reduction, closed lock, scoping review

## Abstract

**Background/Objectives:** Temporomandibular joint (TMJ) mobility disorders encompass opposing clinical problems, ranging from hypermobility, subluxation, and recurrent dislocation to restricted mandibular mobility caused by disc displacement without reduction (DDwoR). Dextrose-based injections have been investigated in both settings, although their therapeutic objectives may differ. This scoping review mapped the clinical indications, injection protocols, outcomes, safety findings, and evidence gaps. **Methods:** PubMed/MEDLINE, Europe PMC, and BASE were searched from inception through 14 June 2026. Study selection and data charting were conducted independently by two reviewers, and primary studies underwent design-specific JBI appraisal. **Results:** Eighteen reports were included: 14 reports describing 13 primary clinical studies (two reports were companion publications from the same randomized pilot cohort) and four systematic reviews used for evidence mapping. Among the primary studies, twelve addressed excessive TMJ mobility, and one evaluated DDwoR with limited mouth opening. In hypermobility and recurrent dislocation, within-group reductions in pain, excessive mouth opening, and recurrence were commonly reported, but superiority over placebo or active comparators was inconsistent. In DDwoR, 5% dextrose was associated with improvements in pain, mouth opening, and chewing function, although the evidence was limited by the small sample. No serious adverse events were reported. **Conclusions:** Substantial heterogeneity precluded quantitative synthesis and prevented identification of an optimal protocol. Indication-specific, adequately powered randomized trials with standardized outcomes and longer follow-up are required.

## 1. Introduction

### 1.1. Rationale

Temporomandibular disorders (TMDs) comprise a heterogeneous group of conditions affecting the temporomandibular joint (TMJ), masticatory muscles, and associated structures. They are common in the general population and may present with orofacial pain, joint sounds, altered mandibular movement, and impaired mandibular function [1,2]. Their etiology and perpetuating factors are multifactorial, resulting in a broad spectrum of clinical presentations [3].

Disturbances of TMJ mobility may occur in opposing directions. Excessive mandibular translation may manifest as symptomatic joint hypermobility, subluxation, or recurrent dislocation [4]. Conversely, disc displacement without reduction may result in closed lock, pain, and restricted mouth opening [5,6]. This distinction is clinically important because treatment goals differ between these conditions. In patients with hypermobility, treatment is intended to improve joint stability and reduce excessive translation or recurrent dislocation, whereas management of disc displacement without reduction aims to relieve pain and restore mandibular mobility and function [4,5,6].

Conservative approaches, including physiotherapy, behavioral interventions, occlusal appliances, and pharmacological treatment, are commonly used in the management of TMDs [2,7]. When symptoms persist or recur, minimally invasive interventions may be considered in appropriately selected patients [4,7]. Intra-articular injections constitute an established component of minimally invasive TMJ treatment, although the available meta-analytic evidence varies across injectable agents, indications, and outcomes [8,9,10]. Emerging biological approaches, including autologous conditioned serum, further illustrate the expanding range of intra-articular therapies; however, the supporting clinical evidence remains preliminary [11]. Dextrose-based injections, frequently described as dextrose prolotherapy, have been investigated in patients with both excessive TMJ mobility and restricted mandibular mobility associated with disc displacement without reduction [5,6,12,13,14,15,16,17,18,19,20,21,22,23].

The proposed biological effects of dextrose prolotherapy include modulation of pain and stimulation of local responses involving inflammatory signaling, fibroblast activation, collagen synthesis, and connective-tissue remodeling [24,25,26,27]. However, these mechanisms have not been conclusively demonstrated in the TMJ and may vary according to dextrose concentration, injection site, and target tissue. Clinical studies have reported changes in pain, mandibular range of motion, joint stability, and the frequency of dislocation or locking episodes, but their findings cannot be interpreted independently of the underlying TMJ condition [5,6,12,13,14,15,16,17,18,19,20,21,22,23,28].

Previous reviews have either focused on individual clinical indications or synthesized broader, heterogeneous TMD populations, potentially obscuring the opposing therapeutic goals associated with excessive and restricted TMJ mobility [28,29,30,31,32]. The present review provides an updated, indication-specific mapping of dextrose-based injections across both ends of the TMJ mobility spectrum, incorporates recent primary evidence, avoids double counting companion reports, and systematically compares injection protocols, clinical outcomes, safety findings, and methodological limitations. This framework offers a more clinically interpretable synthesis and identifies evidence gaps requiring indication-specific investigation.

### 1.2. Objectives

This scoping review aimed to map the evidence on dextrose injections within two predefined TMJ mobility categories: (1) excessive mobility, comprising symptomatic hypermobility, subluxation, and recurrent dislocation, and (2) restricted mobility caused by disc displacement without reduction with limited mouth opening. Within each category, the review examined injection concentration, formulation, anatomical target, technique, treatment schedule, comparators, clinical outcomes, safety, and evidence gaps, with comparative and non-comparative studies synthesized separately.

## 2. Materials and Methods

### 2.1. Protocol and Registration

The protocol was developed a priori but was not registered. Given its evidence-mapping objective, the study was designed as a scoping review and conducted and reported in accordance with the PRISMA extension for scoping reviews (PRISMA-ScR) (Table S1) [33].

### 2.2. Eligibility Criteria

Eligibility criteria were defined a priori. Primary clinical studies were included in the clinical evidence synthesis only if they met all of the following criteria:

- Population: Adolescents or adults with symptomatic TMJ hypermobility, subluxation, recurrent dislocation, joint instability, or disc displacement without reduction associated with restricted mouth opening or closed lock.
- Intervention: Dextrose injection at any concentration or treatment schedule, administered intra-articularly, periarticularly, or to associated soft tissues. Dextrose could be used alone or with a co-intervention, provided that its effects could be distinguished. Variation in concentration, formulation, injection volume, anatomical target, technique, treatment schedule, and co-interventions was permitted because these parameters were prespecified variables for evidence mapping; they were charted for each study and interpreted as distinct protocol characteristics.
- Comparator: A comparator was not required. Eligible comparators included placebo, no treatment, other injectable therapies, physiotherapy, and surgical interventions.
- Outcomes: At least one extractable clinically relevant outcome, including pain intensity, mandibular range of motion, masticatory function, joint stability, frequency of dislocation or locking episodes, time to recurrence, treatment durability, adverse events, complications, or patient-reported outcomes.
- Study design: Randomized or non-randomized clinical trials; controlled or uncontrolled observational studies; cohort or case–control studies; case series; and case reports.
- Publication characteristics: Full text originally published in English, with no restriction on publication year.

Reviews, meta-analyses, and clinical trial protocols were eligible only as secondary sources for evidence mapping and identification of potentially eligible primary studies; they were not included in the clinical evidence synthesis.

Exclusion criteria were as follows:

- Non-human, in vitro, or cadaveric studies.
- Studies of TMD populations without an eligible TMJ mobility disorder, or mixed populations without separately extractable data for an eligible subgroup.
- Studies that did not involve a dextrose injection.
- Studies in which the effects of dextrose could not be distinguished from those of a co-intervention.
- Primary clinical reports without extractable clinically relevant outcomes or clinical data.
- Conference abstracts, letters, editorials, or comments without extractable clinical data.

Reports whose full text could not be obtained after reasonable retrieval attempts were classified separately as reports not retrieved and were not assessed for eligibility.

### 2.3. Information Sources

PubMed (including MEDLINE-indexed records), Europe PMC, and the Bielefeld Academic Search Engine (BASE) were searched from inception through 14 June 2026. These sources were selected to cover peer-reviewed biomedical literature as well as broader scholarly and grey literature relevant to dextrose injections for TMJ mobility disorders.

The reference lists of all included primary studies and relevant narrative reviews, systematic reviews, and meta-analyses were also screened to identify additional eligible publications. When necessary, attempts were made to obtain missing full texts or relevant data by contacting the authors.

### 2.4. Search

A systematic literature search was conducted to identify primary and secondary evidence on dextrose injections for TMJ mobility disorders, including hypermobility, recurrent dislocation, and disc displacement without reduction. The strategy was developed a priori from the predefined eligibility criteria and used a broad, sensitive approach. Search terms were adjusted to the indexing, field structure, and syntax of each database to optimize search sensitivity.

The search strategy combined terms relating to temporomandibular disorders, dextrose, and injection therapy:

(temporomandibular OR “temporomandibular joint” OR “temporomandibular disorder*” OR TMJ OR TMD OR “jaw dislocation” OR “mandibular dislocation”) AND (dextrose OR “hypertonic dextrose” OR prolotherapy OR “dextrose prolotherapy”) AND (injection* OR infiltrat* OR intraarticular OR “intra-articular” OR periarticular).

Phrase searching, truncation, and field restrictions were adapted as supported by PubMed, Europe PMC, and BASE. No date or study-design filters were applied. No language filter was used during database searching; publication language was assessed during full-text eligibility evaluation.

### 2.5. Selection of Sources

All records identified through the electronic searches and supplementary reference-list screening were imported into Rayyan (Rayyan Systems, Inc., Cambridge, MA, USA) [34]. Deduplication was performed by one reviewer (T.H.).

Before formal screening, the reviewers completed a calibration exercise to ensure consistent interpretation and application of the eligibility criteria. Source selection was then conducted in two stages. First, titles and abstracts were screened independently by two reviewers (J.K. and O.J.), with their decisions concealed from each other. Second, the same reviewers independently assessed the full texts of potentially eligible reports against the predefined eligibility criteria.

Disagreements at either stage were resolved through discussion and consensus. Reasons for exclusion following full-text assessment were recorded. Reports for which the full text could not be obtained were classified separately as reports not retrieved. The complete selection process was documented in a flow diagram.

### 2.6. Data Charting Process

Data were charted using a standardized form developed by the review team and pilot-tested on a sample of eligible articles. Any necessary refinements were agreed upon by the reviewers and applied consistently across all included sources. Two reviewers (J.K. and O.J.) independently charted the data in structured tables prepared in Google Sheets (Google LLC., Mountain View, CA, USA). Discrepancies were resolved through discussion and consensus.

### 2.7. Data Items

For primary clinical studies, charted data included author and year, study design, population and diagnosis, sample and group sizes, intervention characteristics, comparator, follow-up duration, outcome measures, main findings, and adverse events. Intervention characteristics included dextrose concentration, injection site and technique, number and timing of treatment sessions, and any co-interventions. Outcomes of interest included pain, mandibular range of motion, joint stability, dislocation or locking episodes, masticatory function, treatment durability, and safety.

For secondary evidence sources, the source type, clinical scope, included evidence base, principal conclusions, relevance to the review, and role in evidence mapping were recorded.

### 2.8. Critical Appraisal of Individual Sources of Evidence

A design-specific critical appraisal of all included primary clinical studies was conducted by one reviewer (K.G.-K.) using the JBI critical appraisal tools [35]. Randomized studies were assessed using the JBI Critical Appraisal Checklist for Randomized Controlled Trials, observational comparative studies using the Checklist for Cohort Studies, and uncontrolled studies using the Checklist for Case Series.

Each applicable item was rated as “Yes”, “No”, “Unclear”, or “Not applicable”. The assessed domains included participant selection, randomization and allocation concealment, baseline comparability, blinding, comparability of treatment, reliability of outcome measurement, confounding, completeness of follow-up, and appropriateness of statistical analysis. No composite quality scores or overall risk-of-bias categories were assigned. The appraisal did not determine study eligibility but was used to contextualize the evidence and inform its narrative interpretation. These design-specific appraisals were used to evaluate risk-of-bias-relevant methodological limitations and to qualify the interpretation of the findings according to study design.

### 2.9. Synthesis of Results

Study characteristics and outcomes were tabulated and synthesized narratively. Primary clinical studies were first grouped by indication: (1) symptomatic TMJ hypermobility, subluxation, or recurrent dislocation and (2) disc displacement without reduction with restricted mouth opening. Within each indication, studies were then stratified as comparative studies, comprising randomized and non-randomized studies with a comparator group, or non-comparative studies, comprising single-arm studies and case series. Between-group findings were reported only for comparative studies. Non-comparative studies were summarized as within-group observations and were not used to infer comparative effectiveness. Injection concentration, formulation, anatomical target, technique, treatment schedule, co-interventions, follow-up, and outcomes were compared descriptively within these strata.

No meta-analysis was performed because of substantial clinical and methodological heterogeneity. Secondary evidence sources were summarized separately for evidence mapping and contextual interpretation and were not combined with primary clinical evidence.

## 3. Results

### 3.1. Selection of Sources of Evidence

Following duplicate removal, records underwent title and abstract screening and subsequent full-text assessment. Inter-reviewer agreement was high at both stages, with Cohen’s κ values of 0.98 for title and abstract screening and 0.82 for full-text assessment. Remaining disagreements were resolved through discussion and consensus.

Eight reports were excluded after full-text assessment because their populations did not meet the predefined eligibility criteria. One additional report could not be retrieved and was not assessed for eligibility. Overall, 18 reports were included: 14 reports representing 13 primary clinical studies and four secondary evidence sources used for evidence mapping. All included reports were originally published in English.

**Figure 1:**
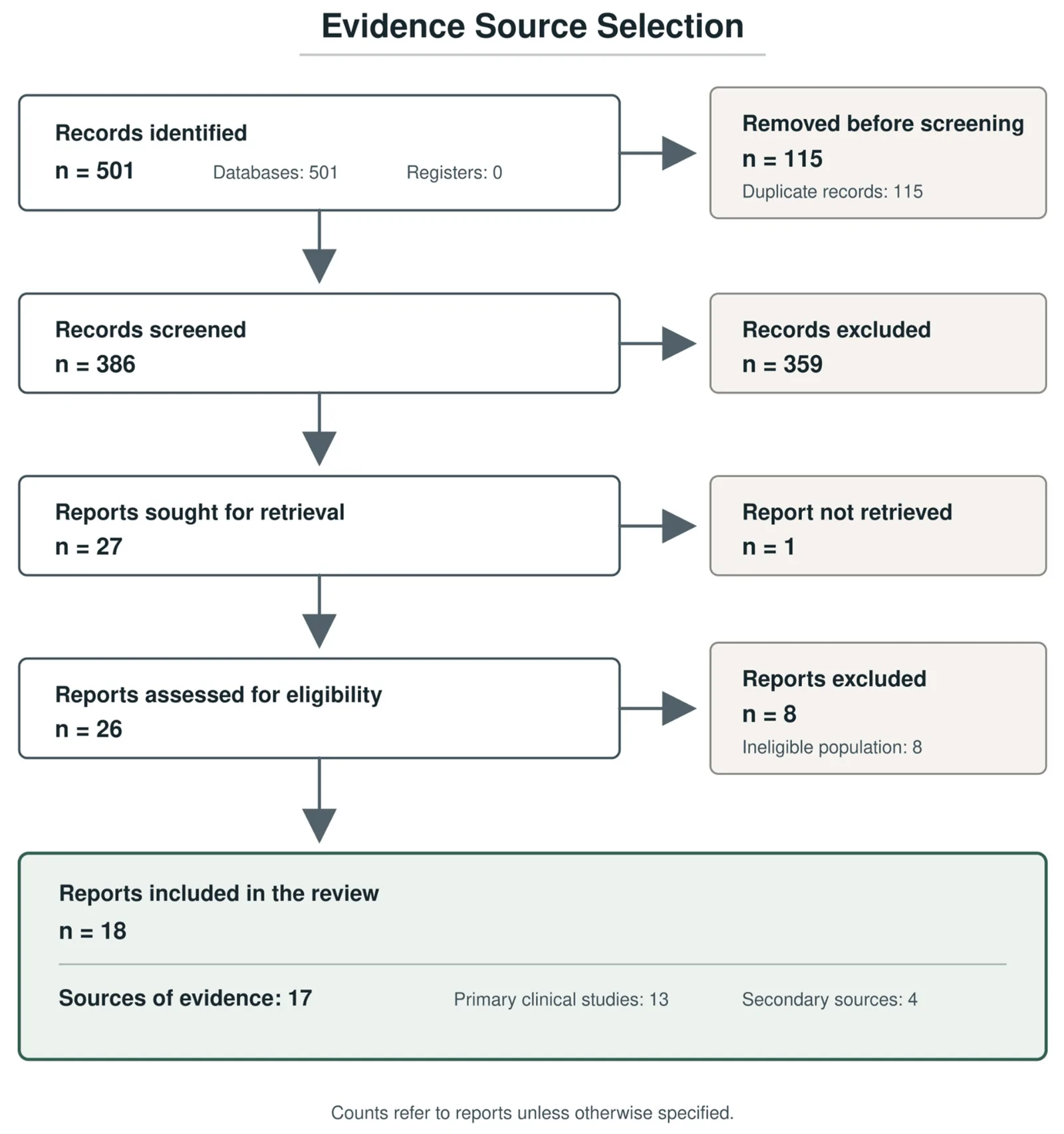
Flow diagram of evidence source identification, screening, eligibility assessment, and inclusion.

Reports excluded after full-text assessment and the reasons for exclusion are presented in Table A1. The complete selection process is shown in the flow diagram (Figure 1).

### 3.2. Characteristics of Sources of Evidence

Fourteen reports representing 13 primary clinical studies were included. Twelve studies investigated dextrose-based injections for excessive TMJ mobility, including hypermobility, subluxation, and recurrent dislocation. One randomized pilot study, reported in two companion publications, evaluated restricted mandibular mobility associated with DDwoR presenting as closed lock with limited mouth opening. The studies varied considerably in design, sample size, injection protocol, comparator, and follow-up duration. General study characteristics are summarized in Table 1, while detailed intervention and comparator protocols are presented in Table 2.

The two publications by Sayed Taleb et al. [5,6] were treated as companion reports from the same randomized pilot cohort to avoid double counting. One report presented pain and maximum interincisal opening outcomes, whereas the other focused on chewing function assessed using the chewing pain index (CPI).

Four systematic reviews were included as secondary sources for evidence mapping and reference checking. Nagori et al. [29] and Abrahamsson et al. [30] were most closely aligned with the target indications because they focused on TMJ hypermobility, subluxation, or luxation. Sit et al. [31] and Saramantos et al. [28] addressed broader and more heterogeneous TMD populations and were therefore used primarily to contextualize the wider evidence on dextrose prolotherapy. Secondary sources were not treated as primary clinical evidence, and their results were not combined with those of individual clinical studies. Their characteristics and roles in the present review are summarized in Table 3.

### 3.3. Critical Appraisal Within Sources of Evidence

The methodological appraisal identified several recurring strengths and limitations across the included studies. Among the randomized trials, randomization procedures were generally described, whereas reporting of allocation concealment and baseline comparability was less consistent. Blinding was absent or insufficiently reported in several trials, particularly when active interventions with visibly different procedures were compared. Follow-up, outcome measurement, and statistical analysis were generally reported adequately, although some studies analyzed only participants who completed follow-up.

The single-arm studies and case series generally provided clear descriptions of the participants, clinical condition, interventions, and outcomes. Their principal limitations concerned the absence of consecutive or complete inclusion of eligible participants and, in some studies, incomplete reporting of the clinical setting.

In the retrospective comparative studies, exposures and outcomes were generally measured consistently between groups. However, none of these studies explicitly identified relevant confounding factors or reported strategies for controlling confounding. In Ates and Sivrikaya, the treatment groups also differed significantly in sex distribution at baseline.

Detailed item-level judgments are presented in Appendix A (Table A2, Table A3 and Table A4). No composite quality scores or overall risk-of-bias categories were assigned, and the appraisal results were not used as eligibility criteria.

### 3.4. Results of Individual Sources of Evidence

The principal clinical, functional, and safety outcomes reported by the included primary studies are summarized in Table 4. Because the studies differed in clinical indication, injection protocol, comparator, outcome definitions, and follow-up duration, their findings are presented descriptively and were not pooled quantitatively.

### 3.5. Synthesis of Results

The narrative synthesis identified two principal clinical indications for dextrose-based injections: excessive TMJ mobility, encompassing hypermobility, subluxation, and recurrent dislocation, and restricted mandibular mobility associated with DDwoR presenting as closed lock. Twelve of the 13 primary studies addressed excessive TMJ mobility [12,13,14,15,16,17,18,19,20,21,22,23], whereas evidence for DDwoR was limited to one randomized pilot study reported in two companion publications [5,6].

Ten studies included comparator groups: seven randomized studies [5,6,12,13,14,17,19,22] and three retrospective comparative studies [15,16,23]. Across these studies, dextrose was associated with improvements within treatment groups, but between-group superiority over placebo or active interventions was inconsistent. Evidence for DDwoR was limited to one small randomized pilot cohort, in which 5% dextrose was associated with improvements in pain, mouth opening, and chewing function [5,6].

Three studies lacked comparator groups [18,20,21]. These studies reported within-group improvements in pain, excessive mouth opening, joint stability, or recurrence. In the absence of a concurrent comparator, these findings were treated solely as descriptive observations and were not interpreted as evidence of treatment efficacy or comparative effectiveness.

Across both design strata, outcome interpretation remained indication-specific. Reduced mouth opening could represent therapeutic stabilization in excessive TMJ mobility, whereas increased mouth opening represented improvement in DDwoR. Serious adverse events were not reported, and the few reported complications were transient. Differences in patient selection, injection protocols, comparators, outcome definitions, and follow-up precluded quantitative pooling.

## 4. Discussion

### 4.1. Summary of Evidence

The findings of this scoping review indicate that the clinical meaning of outcomes following dextrose injections differs according to the underlying TMJ disorder. Most studies of recurrent dislocation, subluxation, or joint hypermobility reported within-group reductions in pain, excessive mouth opening, and the frequency of locking or dislocation episodes [12,13,14,15,16,17,18,19,20,21,22,23]. However, dextrose did not consistently demonstrate superiority over placebo or active comparators. In contrast, evidence for DDwoR with limited mouth opening was restricted to one small, randomized pilot cohort reported in two companion publications. That study found improvements in pain, maximum mouth opening, and chewing function following 5% dextrose injection [5,6]. These findings emphasize that changes in mandibular mobility should be interpreted in relation to the treated condition and should not be generalized across heterogeneous TMD populations.

This indication-specific perspective may help explain some of the variability observed in previous systematic reviews and meta-analyses that evaluated broader TMD populations [25,28,31,32]. By contrast, previous injection-focused syntheses explicitly distinguished recurrent TMJ dislocation from TMJ hypomobility, recognizing that the desired direction of change in mandibular mobility is opposite in these conditions [9,10]. In excessive TMJ mobility, reducing mandibular translation and maximum mouth opening may represent a desirable stabilizing response, whereas restoring mouth opening is a principal therapeutic objective in DDwoR [29,30,32,36]. Distinguishing these opposing treatment goals provides a more clinically meaningful framework for interpreting the available evidence and avoids treating all changes in mandibular mobility as equivalent.

Mechanistic interpretation also requires differentiation between the hypertonic dextrose solutions used in most studies of excessive mobility and the 5% dextrose solution evaluated in DDwoR. Hypertonic dextrose has been proposed to induce a localized inflammatory and reparative response involving fibroblast activation, collagen synthesis, and connective-tissue remodeling, potentially reinforcing periarticular supporting structures [24,25,26,27]. In contrast, 5% dextrose injection is not hypertonic and has been proposed to exert predominantly neurosensory or analgesic effects [5,6,24]. Neither mechanism has been demonstrated directly in the human TMJ. Structural outcomes were assessed infrequently, and the available imaging studies did not establish treatment-related osseous or condylar remodeling [13,18,25].

Interpretation was also guided by study design. The comparative studies did not consistently demonstrate superiority of dextrose over placebo or active comparators. The three non-comparative studies provided only uncontrolled within-group observations, which cannot establish causality or comparative effectiveness. Variation in diagnostic criteria, dextrose concentration, injection site, technique, treatment schedule, comparators, outcome definitions, and follow-up further prevents identification of an optimal injection protocol.

### 4.2. Strengths

This scoping review provides a broad and up-to-date map of the evidence on dextrose-based injections for TMJ disorders characterized by either excessive or restricted mobility. Its principal strength is the indication-specific synthesis, which distinguishes hypermobility, subluxation, and recurrent dislocation from DDwoR with limited mouth opening. This distinction enables clinically meaningful interpretation of mandibular mobility outcomes, because reduced movement may represent therapeutic stabilization in hypermobility, whereas increased mouth opening is desirable in DDwoR. The review also incorporates recently published evidence, avoids double counting companion reports from the same cohort, and systematically maps variation in patient selection, injection protocols, comparators, outcomes, and follow-up. Finally, the item-level methodological appraisal provides additional transparency regarding the strengths and limitations of the included studies.

### 4.3. Limitations

The evidence base was limited by substantial clinical and methodological heterogeneity in patient selection, diagnostic criteria, dextrose concentration, injection site, number of treatment sessions, comparators, outcome definitions, and follow-up duration [25,28,31,32,37]. Most studies enrolled small samples, and several used uncontrolled, retrospective, or incompletely blinded designs. Outcomes and definitions of treatment success were not standardized, limiting robust direct comparisons and preventing identification of an optimal injection protocol. Evidence for DDwoR was particularly limited, originating from a single pilot cohort reported in two companion publications.

Limitations of the review process should also be acknowledged. Although the search included biomedical and grey-literature sources, it was restricted to three electronic platforms and may not have identified all relevant reports. Restriction to English-language publications may have introduced language bias and excluded relevant evidence published in other languages. One potentially eligible report could not be retrieved and therefore could not be assessed. The protocol was not prospectively registered, and the methodological appraisal was conducted by one reviewer. Finally, the indication-specific eligibility criteria excluded broadly defined TMD populations when results for eligible mobility disorders were not separately extractable. This improved clinical specificity but may have omitted indirect evidence relevant to dextrose injections in the wider TMD population.

### 4.4. Recommendations for Future Research

Future research should examine excessive and restricted TMJ mobility as separate clinical indications. For hypermobility, subluxation, and recurrent dislocation, adequately powered multicenter randomized trials should compare dextrose with placebo and clinically relevant active treatments, including autologous blood injection, botulinum toxin, arthrocentesis, and combined strategies. Evidence for DDwoR remains limited to one small pilot cohort, making independent confirmation in larger trials a priority. Eligibility should be based on standardized diagnostic criteria, and findings from controlled and uncontrolled designs should be interpreted separately.

Intervention protocols should consistently report the prepared and final dextrose concentration, injection volume, diluent or local anesthetic, anatomical target, injection technique, use of imaging guidance, treatment schedule, and co-interventions. Outcome selection should reflect indication-specific therapeutic goals, including recurrence, open-locking episodes, and joint stability in excessive mobility, and restoration of mouth opening, pain reduction, and chewing function in DDwoR. Validated patient-reported measures, prespecified thresholds for clinically meaningful improvement, longer follow-up, and systematic adverse-event reporting are also needed. Mechanistic and imaging studies could clarify whether 5% and hypertonic dextrose exert distinct biological effects and whether clinical improvement is accompanied by structural changes.

## 5. Conclusions

Dextrose-based injections may provide clinical benefits in selected TMJ mobility disorders, but the available evidence remains limited and heterogeneous. In hypermobility, subluxation, and recurrent dislocation, reductions in pain, excessive mandibular mobility, and recurrence were commonly reported, although comparative superiority over placebo or active treatments was inconsistent. Evidence for 5% dextrose injections in DDwoR with limited mouth opening was confined to one small pilot cohort. Current data are insufficient to establish an optimal injection protocol or support definitive conclusions regarding comparative effectiveness and safety. Adequately powered, indication-specific randomized trials with standardized protocols, validated outcomes, and longer follow-up are required.

## Figures and Tables

**Table 1:** General characteristics of the included primary clinical studies evaluating dextrose injections for TMJ mobility disorders.

Author, Year	Study Design	Clinical Indication and Population	Sample Size and Groups	Follow-Up
Sayed Taleb et al., 2025a,b [5,6]	Pilot randomized controlled trial reported in two publications	Adult women with unilateral DDwoR presenting as closed lock with limited mouth opening	20: D5W, *n* = 10; saline, *n* = 10	2 weeks and 2, 6, and 12 months
Ates and Sivrikaya, 2025 [23]	Retrospective comparative cohort study	Patients with unilateral or bilateral symptomatic TMJ hypermobility, open locking, joint sounds, and facial pain	26 analyzed: combined treatment, *n* = 15; prolotherapy alone, *n* = 11	1 week and 1 and 3 months
Chhapane et al., 2024 [12]	Prospective randomized clinical trial	Adults with chronic recurrent unilateral or bilateral TMJ dislocation	32: dextrose, *n* = 16; ABI, *n* = 16	3 days; 1 and 2 weeks; and 1, 3, 6, and 12 months
Bhargava et al., 2023 [13]	Preliminary randomized clinical study	Patients with chronic symptomatic and painful TMJ hypermobility or subluxation	60: HDP, *n* = 30; ABI, *n* = 30	6 and 12 months
Çömert Kiliç et al., 2023 [14]	Prospective randomized clinical trial	Adults with symptomatic TMJ subluxation, including open-locking episodes and facial pain	30 enrolled; 25 analyzed: dextrose, *n* = 14; botulinum toxin, *n* = 11	8–12 months
Pandey et al., 2022 [15]	Retrospective comparative cohort study	Patients with chronic recurrent bilateral TMJ dislocation	20: dextrose, *n* = 10; ABI, *n* = 10	1 and 2 weeks and 1, 3, and 6 months
Taşkesen and Cezairli, 2023 [16]	Retrospective comparative clinical study	Patients with symptomatic TMJ hypermobility, including subluxation, open locking, and functional pain	24: prolotherapy, *n* = 12; combined treatment, *n* = 12	Short- and longer-term assessments during and after treatment
Mustafa et al., 2018 [17]	Prospective randomized clinical trial	Patients with bilateral symptomatic TMJ hypermobility, including subluxation or dislocation	40 enrolled; 37 analyzed: control, *n* = 9; 10% dextrose, *n* = 10; 20% dextrose, *n* = 9; 30% dextrose, *n* = 9	Monthly assessments; final assessment at approximately 4 months
Refai, 2017 [18]	Prospective single-arm study	Patients with bilateral symptomatic TMJ hypermobility, pain, locking, and abnormal condylar excursion	61	During treatment, 3 months after treatment, and long-term follow-up of 1–4 years
Çömert Kiliç and Güngörmüş, 2016 [19]	Prospective randomized clinical trial	Adults with bilateral symptomatic TMJ hypermobility, open locking, joint sounds, and facial pain	30 enrolled; 26 analyzed: dextrose, *n* = 14; placebo, *n* = 12	12 months after the third injection
Zhou et al., 2014 [20]	Prospective case series	Younger patients with non-neurogenic recurrent TMJ dislocation	45	4 weeks and 3 and 6 months; subsequent telephone follow-up, mean 18 months
Ungor et al., 2013 [21]	Retrospective case series	Women with acute or chronic TMJ dislocation or luxation and a history of locking episodes	10	Assessments during treatment at 6-week intervals; final assessment at 24 weeks
Refai et al., 2011 [22]	Prospective randomized, double-blind, placebo-controlled trial	Patients with bilateral symptomatic TMJ hypermobility, painful subluxation or dislocation, and open-locking episodes	12: dextrose, *n* = 6; placebo, *n* = 6	Every 6 weeks during treatment and 3 months after the final injection

Abbreviations: ABI, autologous blood injection; D5W, 5% dextrose in water; DDwoR, disc displacement without reduction; HDP, heavy bupivacaine–dextrose prolotherapy; TMJ, temporomandibular joint.

**Table 2:** Intervention and comparator protocols in the included primary clinical studies.

Author, Year	Dextrose Formulation	Injection Target and Technique	Treatment Schedule and Co-Interventions	Comparator
Sayed Taleb et al., 2025a,b [5,6]	5% dextrose in water	Injection into the retrodiscal tissues	Single treatment session	0.9% saline administered using the same approach
Ates and Sivrikaya, 2025 [23]	20% dextrose	TMJ prolotherapy; the combined-treatment group additionally underwent arthrocentesis with Ringer’s solution	Single treatment session	20% dextrose prolotherapy alone
Chhapane et al., 2024 [12]	3 mL of 50% dextrose	Following TMJ lavage, 2 mL was injected into the superior joint space and 1 mL into the pericapsular tissues	Single session; elastic bandage, soft diet, and subsequent rehabilitation exercises	3 mL of autologous blood administered at the same sites after lavage
Bhargava et al., 2023 [13]	3 mL of heavy bupivacaine–dextrose solution; final dextrose concentration approximately 8%	Following joint lavage, 1 mL was injected into the superior joint space and 2 mL into the pericapsular and retrodiscal tissues	Up to four sessions at 6-week intervals, according to clinical response	3 mL of autologous blood administered to the same anatomical regions
Çömert Kiliç et al., 2023 [14]	5 mL solution containing 2 mL of 30% dextrose, 2 mL of saline, and 1 mL of 2% local anesthetic; final dextrose concentration 12%	Posterior disc attachment, superior joint space, superior and inferior capsular attachments, and stylomandibular ligament; 1 mL per site	Three sessions at 1-month intervals	Single botulinum toxin type A injection into the lateral pterygoid muscles
Pandey et al., 2022 [15]	3 mL of 25% dextrose	Bilateral injections: 2 mL into the superior joint space and 1 mL into the pericapsular tissues	Single session; bandage for 1 week and soft diet for 2 weeks	3 mL of autologous blood administered using the same distribution
Taşkesen and Cezairli, 2023 [16]	10% dextrose	Intra- and periarticular TMJ injections	Three sessions at 4-week intervals	The same prolotherapy protocol combined with one arthrocentesis session
Mustafa et al., 2018 [17]	Final injectable dextrose concentrations of 5%, 10%, or 15%, prepared by mixing 10%, 20%, or 30% dextrose with an equal volume of 2% lidocaine; 3 mL per session	Superior joint space, posterior disc attachment, and superior and inferior capsular attachments	Four sessions at 1-month intervals	Saline with lidocaine administered at the same sites and intervals
Refai, 2017 [18]	3 mL dextrose–mepivacaine solution	Intra-articular and pericapsular injections	Four sessions at 6-week intervals	None
Çömert Kiliç and Güngörmüş, 2016 [19]	5 mL solution containing 2 mL of 30% dextrose, 2 mL of saline, and 1 mL of 2% local anesthetic; final dextrose concentration 12%	Posterior disc attachment, superior joint space, superior and inferior capsular attachments, and stylomandibular ligament; 1 mL per site	Three sessions at 1-month intervals	Saline and local anesthetic administered at the same sites and intervals
Zhou et al., 2014 [20]	50% dextrose with lignocaine	Single posterior periarticular injection site, without entering the joint space	One to three sessions according to clinical response	None
Ungor et al., 2013 [21]	10% dextrose	TMJ prolotherapy injections	Sessions performed at 6-week intervals according to clinical response	None
Refai et al., 2011 [22]	3 mL solution prepared from 2 mL of 10% dextrose and 1 mL of 2% mepivacaine; final dextrose concentration approximately 6.7%	Superior joint space and superior and inferior capsular attachments	Four sessions at 6-week intervals	Saline–mepivacaine solution administered at the same sites and intervals

Abbreviations: ABI, autologous blood injection; TMJ, temporomandibular joint.

**Table 3:** Secondary evidence sources included for evidence mapping.

Author, Year	Type of Source	Scope	Included Evidence	Role in the Present Review
Saramantos et al., 2025 [28]	Systematic review and meta-analysis	Prolotherapy for a broad range of TMDs	12 RCTs	Provided an updated overview of randomized evidence concerning pain, mandibular mobility, mouth opening, clicking, and locking episodes.
Sit et al., 2021 [31]	Systematic review and meta-analysis	Hypertonic dextrose prolotherapy for TMD and TMJ dysfunction	10 RCTs	Contextualized the broader evidence concerning pain, dysfunction, disability, and mouth opening.
Abrahamsson et al., 2020 [30]	Systematic literature review	Surgical and nonsurgical treatment of acute and recurrent TMJ luxation	8 RCTs: 3 on manual reduction and 5 on minimally invasive injection treatments	Supported identification and contextualization of injection-based treatments for recurrent luxation, including dextrose prolotherapy and ABI.
Nagori et al., 2018 [29]	Systematic review and meta-analysis	Dextrose prolotherapy versus placebo for TMJ hypermobility	3 RCTs	Provided indication-specific evidence concerning pain, maximum mouth opening, and recurrence of subluxation or dislocation.

Abbreviations: ABI, autologous blood injection; RCT, randomized controlled trial; TMD, temporomandibular disorder; TMJ, temporomandibular joint.

**Table 4:** Outcome measures and principal findings of the included primary clinical studies.

Author, Year	Main Outcomes	Main Findings
Comparative studies		
Sayed Taleb et al., 2025a,b [5,6]	Pain intensity, MIO, chewing function, CPI	Pain decreased in both groups, with greater improvement after D5W than after saline through 6 months; the between-group difference was no longer significant at 12 months. MIO increased in both groups, with a greater improvement in the D5W group at 12 months. CPI decreased in both groups and was significantly lower after D5W at 2 weeks and 2 and 6 months.
Ates and Sivrikaya, 2025 [23]	MIO, pain, clicking, adverse events	MIO decreased significantly in both groups by 3 months. Pain decreased in both groups, with a greater reduction after combined arthrocentesis and prolotherapy than after prolotherapy alone. Clicking decreased without a significant between-group difference. Transient adverse events resolved spontaneously.
Chhapane et al., 2024 [12]	Pain, joint hypermobility, maximum mouth opening, clicking or crepitus, dislocation frequency, repeated injections, adverse events	Both ABI and dextrose reduced pain and joint hypermobility. ABI produced a greater reduction in mouth opening and fewer subsequent dislocations at longer follow-up. Repeated injections were effective when required, and no major adverse events were reported.
Bhargava et al., 2023 [13]	Pain, mouth opening, CT and MRI findings, repeated injections, adverse events	Both HDP and ABI reduced pain and mouth opening. No treatment-related condylar morphological changes or major complications were identified. HDP produced clinical outcomes broadly comparable to those of ABI.
Çömert Kiliç et al., 2023 [14]	Locking frequency, patient satisfaction	Locking frequency decreased significantly in both groups, without a significant difference between botulinum toxin type A and dextrose prolotherapy. At final follow-up, 8 of 14 patients receiving prolotherapy (57.1%) reported no locking episodes.
Pandey et al., 2022 [15]	Maximum mouth opening, pain, dislocation frequency, laterotrusion, protrusion, TMJ sounds	Both interventions improved clinical outcomes. Dextrose produced greater pain reduction, whereas ABI produced greater reductions in mouth opening and mandibular excursions. The groups did not differ significantly in dislocation frequency or TMJ sounds.
Taşkesen and Cezairli, 2023 [16]	Maximum and painless mouth opening, pain at rest, pain during chewing, TMJ sounds, locking frequency	Pain decreased significantly in both groups. Maximum mouth opening decreased significantly in the prolotherapy-only group, whereas locking frequency improved to a greater extent after prolotherapy combined with arthrocentesis. No serious adverse events were reported.
Mustafa et al., 2018 [17]	Pain, maximum mouth opening, joint sounds, open-locking or luxation episodes, adverse events	Pain and maximum mouth opening decreased over time in all groups, including the control group. Joint sounds also decreased, and no open-locking or luxation episodes were reported during follow-up. No significant differences were found between the control group and the different dextrose concentrations.
Çömert Kiliç and Güngörmüş, 2016 [19]	Masticatory efficiency, pain, joint sounds, mouth opening, mandibular movements, adverse events	Masticatory efficiency, pain, and joint sounds improved in both groups. MIO decreased significantly only in the dextrose group, but no significant between-group differences were identified. Dextrose prolotherapy was not superior to saline placebo.
Refai et al., 2011 [22]	Pain on palpation, maximum mouth opening, clicking, locking or luxation episodes, adverse events	Pain and locking frequency decreased in both groups, without significant between-group differences. Maximum mouth opening decreased significantly only in the dextrose group, and no participants in that group reported locking at final follow-up. No serious adverse events were reported.
Non-comparative studies		
Refai, 2017 [18]	Pain during function, MIO, clicking, locking frequency, tomographic findings, adverse events	Pain, locking frequency, and MIO decreased significantly over time, and clicking became less frequent. At long-term follow-up, most patients reported no locking or pain. Tomographic assessment showed no treatment-related osseous changes.
Zhou et al., 2014 [20]	Recurrent dislocation or subluxation, clicking, maximum mouth opening, number of injections, adverse events	The reported treatment success rate was 91%, with 41 of 45 patients experiencing no further dislocation or subluxation for more than 6 months. Dislocation frequency and clicking decreased, whereas mouth opening did not change significantly. No serious adverse events were reported.
Ungor et al., 2013 [21]	Locking frequency, maximum mouth opening, clicking, pain during function, chewing function, quality of life, adverse events	Locking episodes decreased after the first injection and were absent between weeks 12 and 24. Pain during function and quality of life improved significantly. Mouth opening decreased without reaching statistical significance, and clicking resolved in most patients after the first session. No serious adverse events were reported.

Abbreviations: ABI, autologous blood injection; CPI, chewing pain index; CT, computed tomography; D5W, 5% dextrose in water; HDP, heavy bupivacaine–dextrose prolotherapy; MIO, maximum interincisal opening; MRI, magnetic resonance imaging; TMJ, temporomandibular joint.

## Data Availability

The data supporting the findings of this review are contained within the article and Appendix A. The data-charting form and extracted dataset are available from the corresponding author upon reasonable request.

## Supplementary Materials

The following supporting information can be downloaded at: https://www.mdpi.com/article/10.3390/jcm15176708/s1, Table S1: PRISMA 2020 Checklist.

## Appendix A

**Table A1:** Reports excluded after full-text assessment and reasons for exclusion.

Title	Author, Year	Reason for Exclusion
The Effectiveness of Ultrasound-Guided Temporomandibular Joint Injection in Reduction of Complications Rate Associated With 50% Hypertonic Prolotherapy: Comparative Study	Hassan et al., 2026 [38]	Ineligible study population: patients with broadly defined temporomandibular joint internal derangement; no eligible mobility disorder or separately extractable eligible subgroup was reported.
Short Term Clinical Outcomes of Ultrasound-Guided Injection of 50% Hypertonic Prolotherapy in Temporomandibular Joint Internal Derangement: Comparative Study	Mohammed and Hassan, 2026 [39]	Ineligible study population: patients with broadly defined temporomandibular joint internal derangement; no eligible mobility disorder or separately extractable eligible subgroup was reported.
Efficacy of Prolotherapy for Temporomandibular Joint Dysfunction: An Interventional Clinical Study	Assiri et al., 2025 [40]	Ineligible study population: general temporomandibular disorder cohort without diagnosis-specific or separately extractable data for an eligible mobility disorder.
Evaluation of the efficacy of autologous conditioned serum versus dextrose prolotherapy in internal derangement of the TMJ—A pilot study	Ravikumar et al., 2024 [41]	Ineligible study population: mixed internal derangement population comprising Wilkes stages II–V; outcomes were not reported separately for an eligible mobility disorder.
Evaluation of Efficacy of 10% Dextrose Prolotherapy in Management of Temporomandibular Joint Disorders: A Prospective Study	Singh et al., 2024 [42]	Ineligible study population: general temporomandibular disorder cohort without diagnosis-specific or separately extractable data for an eligible mobility disorder.
Dextrose Prolotherapy Versus Lidocaine Injection for Temporomandibular Dysfunction: A Pragmatic Randomized Controlled Trial	Zarate et al., 2020 [43]	Ineligible study population: broadly defined temporomandibular disorder population without an eligible mobility disorder or separately extractable eligible subgroup.
Treatment of Temporomandibular Dysfunction With Hypertonic Dextrose Injection (Prolotherapy): A Randomized Controlled Trial With Long-term Partial Crossover	Louw et al., 2019 [44]	Ineligible study population: broadly defined temporomandibular disorder population without an eligible mobility disorder or separately extractable eligible subgroup.
Change of site of intra-articular injection of hypertonic dextrose resulted in different effects of treatment	Fouda, 2018 [45]	Ineligible study population: disc displacement with reduction, clicking, and pain with preserved mouth opening; no eligible mobility disorder was investigated.

**Table A2:** JBI critical appraisal of included randomized controlled trials.

JBI Item	Sayed Taleb et al., 2025a,b [5,6]	Chhapane et al., 2024 [12]	Bhargava et al., 2023 [13]	Çömert Kiliç et al., 2023 [14]	Mustafa et al., 2018 [17]	Çömert Kiliç and Güngörmüş, 2016 [19]	Refai et al., 2011 [22]
True randomization?	Yes	Yes	Yes	Yes	Yes	Yes	Yes
Allocation concealed?	Yes	Unclear	Unclear	Yes	Yes	Yes	Yes
Groups similar at baseline?	Yes	Unclear	Unclear	Yes	Unclear	Yes	Yes
Participants blinded?	Yes	No	No	No	No	No	Yes
Treatment providers blinded?	Yes	No	No	No	No	No	Yes
Outcome assessors blinded?	Yes	Yes	Yes	Yes	Yes	Yes	Yes
Identical treatment apart from intervention?	Yes	Yes	Yes	Yes	Yes	Yes	Yes
Follow-up complete or adequately addressed?	Yes	Yes	Yes	Yes	Yes	Yes	Yes
Analysis by randomized group?	Yes	Yes	Yes	Yes	Yes	Yes	Yes
Outcomes measured similarly between groups?	Yes	Yes	Yes	Yes	Yes	Yes	Yes
Outcomes measured reliably?	Yes	Yes	Yes	Yes	Yes	Yes	Yes
Appropriate statistical analysis?	Yes	Yes	Yes	Yes	Yes	Yes	Yes
Appropriate trial design and handling of deviations?	Yes	Yes	Yes	Yes	Yes	Yes	Yes

Abbreviations: JBI, Joanna Briggs Institute.

**Table A3:** JBI critical appraisal of included case series and single-arm studies.

JBI Item	Refai, 2017 [18]	Zhou et al., 2014 [20]	Ungor et al., 2013 [21]
Clear inclusion criteria?	Yes	Yes	Yes
Standardized measurement of condition?	Yes	Yes	Yes
Valid identification of condition?	Yes	Yes	Yes
Consecutive inclusion?	Yes	No	No
Complete inclusion?	Yes	No	No
Clear participant demographics?	Yes	Yes	Yes
Clear clinical information?	Yes	Yes	Yes
Clear reporting of outcomes and follow-up?	Yes	Yes	Yes
Clear reporting of site or clinic demographics?	Yes	Yes	No
Appropriate statistical analysis?	Yes	Yes	Yes

Abbreviations: JBI, Joanna Briggs Institute.

**Table A4:** JBI critical appraisal of included comparative cohort studies.

JBI Item	Ates and Sivrikaya, 2025 [23]	Pandey et al., 2022 [15]	Taşkesen and Cezairli, 2023 [16]
Comparable groups from the same population?	No	Yes	Yes
Similar exposure classification between groups?	Yes	Yes	Yes
Valid and reliable exposure measurement?	Yes	Yes	Yes
Confounding factors identified?	No	No	No
Strategies for controlling confounding reported?	No	No	No
Outcome absent at baseline?	Yes	Yes	Yes
Valid and reliable outcome measurement?	Yes	Yes	Yes
Sufficient follow-up duration?	Yes	Yes	Yes
Follow-up complete or losses adequately explained?	Yes	Yes	Yes
Strategies for addressing incomplete follow-up?	NA	NA	NA
Appropriate statistical analysis?	No	Yes	Yes

Abbreviations: JBI, Joanna Briggs Institute; NA, not applicable.
